# Mindfulness-Based Cognitive Therapy for Improving Subjective Well-Being Among Healthy Individuals: Protocol for a Randomized Controlled Trial

**DOI:** 10.2196/15892

**Published:** 2020-05-08

**Authors:** Mitsuhiro Sado, Teppei Kosugi, Akira Ninomiya, Maki Nagaoka, Sunre Park, Daisuke Fujisawa, Joichiro Shirahase, Masaru Mimura

**Affiliations:** 1 Department of Neuropsychiatry Keio University School of Medicine Tokyo Japan; 2 Faculty of Nursing and Medicine Care Keio University Tokyo Japan

**Keywords:** mindfulness-based cognitive therapy, subjective well-being, healthy individuals, randomized controlled trial, cost-effectiveness

## Abstract

**Background:**

Previous studies have indicated that higher subjective well-being works as a protective factor for health. Some studies have already shown the effects of mindfulness-based interventions on improving subjective well-being. However, these studies targeted specific populations rather than the general public. Furthermore, they assessed either life evaluation or affective aspects of subjective well-being rather than the concept as a whole, including the eudemonic aspect of well-being.

**Objective:**

This study aims to investigate the effectiveness and cost-effectiveness of mindfulness-based cognitive therapy (MBCT) for improving the wholistic aspects of subjective well-being in healthy individuals.

**Methods:**

This study was an 8-week, randomized, parallel-group, superiority trial with a 2-month follow-up. Healthy individuals aged 20-65 years with scores lower than 25 on the Satisfaction With Life Scale (SWLS) were eligible to participate and randomly allocated to the MBCT group or the wait-list control group. The intervention program was developed by modifying an MBCT program to improve the well-being of a nonclinical population. The primary outcome was the difference between the two groups in mean change scores from the baseline on the SWLS. The secondary outcomes included scores on the Flourishing Scale and the Scale of Positive and Negative Experience as well as the incremental cost-effectiveness ratio.

**Results:**

This study began recruiting participants in July 2018 and recruitment was completed at the end of September 2019. Data collection and dataset construction was completed by the end of March 2020.

**Conclusions:**

This study is unique in that it investigates MBCT’s effects on the three different aspects of subjective well-being: life evaluation, affect, and eudemonia. It is limited, as the specific effect attributable to MBCT cannot be detected because of the lack of an active control group.

**Trial Registration:**

University Hospital Medical Information Network Clinical Trials Registry (UMIN-CTR) UMIN000031885; https://upload.umin.ac.jp/cgi-open-bin/ctr_e/ctr_view.cgi?recptno=R000036376

**International Registered Report Identifier (IRRID):**

DERR1-10.2196/15892

## Introduction

### Background

Subjective well-being has become a central issue in the development of public policy. In this context, there are concerns about the adequacy of current measures of economic performance, such as gross domestic product (GDP), to indicate societal well-being [1]. The Organisation of Economic Co-operation and Development (OECD) proposed that subjective well-being should be considered in addition to these objective scales as a complementary indicator of people’s well-being [2]. Following this initiative, several countries have launched challenges to propose a substitute indicator that complements GDP by measuring the progress of society [1,3-7].

Although arguments about the definition of subjective well-being are still in progress, the most widely accepted one is “good mental states, including all of the various evaluations, positive and negative, that people make of their lives, and the affective reactions of people to their experiences” [2]. There is a general consensus among experts that subjective well-being consists of at least two aspects: life evaluation and affect. In addition, several researchers have insisted that the eudemonic aspect, reflecting people’s sense of purpose and engagement, should also be included in subjective well-being [8]. Thus, subjective well-being consists of three dimensions: life evaluation, affect, and eudemonia.

The importance of subjective well-being is not limited to economics. Several studies have indicated that subjective well-being affects the health of the general public. Steptoe et al revealed that impairment of subjective well-being by depression and life stress elevates the risk of premature death [9]. In addition, higher eudemonic well-being may work as a protective factor for health [10-12]. Therefore, improving the subjective well-being of the general public is significant from a public health perspective.

### What We Already Know

Several interventions, such as a positive events diary [13], life coaching and attainment of goals [14], and positive future thinking [15], have proven effective in the improvement of subjective well-being for nonclinical populations. Furthermore, mindfulness-based intervention (MBI) is another measure that potentially improves people’s subjective well-being. Although MBI was originally developed for the treatment of clinical populations, such as patients with chronic pain [16], depression [17], or anxiety disorders [18], its scope has recently expanded to nonclinical populations. Some studies have already shown its effects on decreasing stress and improving subjective well-being [19-30].

### Rationale for the Study

The studies discussed above, however, have several limitations. First, because they tended to target specific populations, such as students [20,22,26,28,29], schoolteachers [21,23], health care professionals [19,27], and workers in the workplace [31-34], the generalizability of the results to the community is limited. Second, although two studies targeted healthy individuals in the community [24,25], they assessed either the life evaluation or affective aspect of subjective well-being rather than all three aspects (ie, cognitive, affective, and eudemonic aspects). Thus, no study has evaluated the eudemonic aspect of well-being, which has been proven to have a relationship with health [35]. Finally, although the effect of mindfulness-based stress reduction (MBSR) on subjective well-being has been evaluated, no study has assessed the effect of mindfulness-based cognitive therapy (MBCT) [36], which is the other major MBI currently practiced. Therefore, we decided to conduct a randomized controlled trial to demonstrate MBCT’s effectiveness on three different aspects of subjective well-being (ie, life evaluation, affect, and eudemonia) for healthy individuals sampled from community residents.

### Aim

The primary objective of this study is to investigate the effectiveness and cost-effectiveness of MBCT for improving the subjective well-being of healthy individuals in a randomized, wait-list, and controlled trial.

## Methods

### Participants

The study is being conducted at Keio University Hospital in Tokyo, Japan. Participants will be recruited through the Center for Stress Research at Keio University (Keio CSR). Eligible participants are people (1) between the ages of 20 and 65 years, (2) without a history of psychiatric disorders or who have been recovered from psychiatric disorders for more than 2 years, (3) with scores lower than 25 on the Satisfaction With Life Scale (SWLS), and (4) who can provide written informed consent.

Participants will be excluded if they (1) are difficult to follow up with 4 months after the start of the intervention, (2) have a past history of MBIs equivalent to the program provided in the study, and (3) have severe physical conditions.

### Enrollment

Prospective participants who apply to the study through the form at the Keio CSR’s website will be asked to fill out screening questionnaires via the Web (ie, the first screening). If the participants pass the first screening, they will meet a member of the study team who will conduct a face-to-face interview (ie, the second screening) to establish if they meet the inclusion criteria. The Japanese version of the Structured Clinical Interview for the Diagnostic and Statistical Manual of Mental Disorders, Fourth Edition (DSM-IV), Axis I Disorders [37], will be used for diagnostic assessment. The first, second, and third authors (MS, TK, and AN) will conduct the second screening. The eligibility of the participants will be judged based on the results of this second screening. All participants will provide written informed consent after receiving a detailed explanation of all the procedures and will be able to withdraw their consent at any time without negative consequences.

### Baseline Assessment

#### Overview

The participants will complete a battery of questionnaires assessing demographic and psychosocial data. Psychological measures to be obtained will include the SWLS, the Flourishing Scale (FS), the Scale of Positive and Negative Experience (SPANE), the Rosenberg Self-Esteem Scale (RSES), the Five Facet Mindfulness Questionnaire (FFMQ), the Connor-Davidson Resilience Scale (CD-RISC), the Self-Compassion Scale (SCS), the 16-item Quick Inventory of Depressive Symptomatology (QIDS), the Generalized Anxiety Disorder 7-item scale (GAD-7), the Perceived Stress Scale (PSS), the World Health Organization Health and Work Performance Questionnaire (WHO-HPQ), the Multidimensional Assessment of Interoceptive Awareness (MAIA), and the European Quality of Life Five-Dimension Five-Level Scale (EQ-5D-5L). All measures have been validated in Japan [38-49]. The details of each scale are described below.

#### Satisfaction With Life Scale

The SWLS is a self-reported questionnaire with five questions. The scale focuses particularly on assessing one’s life satisfaction. Total scores range from 5 to 35, with higher scores indicating higher satisfaction [50].

#### Flourishing Scale

The FS consists of eight items describing important aspects of human functioning, ranging from positive relationships to feelings of competence, meaning, and purpose in life. Each item is answered on scale ranging from 1 (*strong disagreement*) to 7 (*strong agreement*). Total scores can range from 8 (*strong disagreement* with all items) to 56 (*strong agreement* with all items). Although the scale does not provide separate measures for distinct facets of well-being, it does yield an overview of positive functioning across diverse domains that are widely believed to be important [51].

#### Scale of Positive and Negative Experience

The SPANE measure is a brief 12-item scale with six items devoted to positive experiences and six items designed to assess negative experiences. Because the scale includes general positive and negative feelings, it assesses the full range of positive and negative experiences, including specific feelings that may have unique labels in particular cultures [51].

#### Rosenberg Self-Esteem Scale

The RSES was developed as a brief self-rated assessment to determine self-esteem, self-worth, acceptability, and confidence. It comprises 10 items that allow four responses on a Likert scale, ranging from 1 (*strongly disagree*) to 4 (*strongly agree*). Total possible scores range from 10 to 40; higher scores represent higher self-esteem [52].

#### Five Facet Mindfulness Questionnaire

The FFMQ is a self-report questionnaire used to assess mindfulness ability. It consists of five factors, which were designed based on a factor analytic study of five independently developed mindfulness questionnaires. The five facets are observing, describing, acting with awareness, not judging inner experience, and not reacting to inner experience [53].

#### Connor-Davidson Resilience Scale

The CD-RISC was developed as a brief self-rated assessment to help quantify resilience. The scale contains 25 items, all of which feature a 5-point range of responses, ranging from 0 (*not true at all*) to 4 (*true nearly all of the time*). The total score ranges from 0 to 100, with higher scores reflecting greater resilience [54].

#### Self-Compassion Scale

The SCS assesses individuals’ ability to be kind and understanding toward themselves as opposed to harsh and self-critical in instances of pain or failure. It consists of 29 items and generates scores on six subscales: self-kindness, self-judgment, common humanity, isolation, mindfulness, and overidentification. Participants’ responses are based on the frequency of certain thoughts and feelings. Total subscale scores range from 1 to 5, with higher scores indicating more self-compassion [55].

#### 16-Item Quick Inventory of Depressive Symptomatology

The QIDS is one of the most widely used self-reported questionnaires assessing depressive symptoms. The scoring system for the QIDS converts responses to 16 separate items into the nine DSM-IV symptom criterion domains. Total scores range from 0 to 27. Higher scores indicate higher levels of depressive symptoms [56].

#### Generalized Anxiety Disorder 7-Item Scale

The GAD-7, a 7-item questionnaire, was developed by asking patients how often, during the preceding 2 weeks, they had experienced a set of symptoms. There were four response options on a Likert scale, ranging from 0 (*not at all*) to 3 (*nearly every day*). Scores range from 0 to 21, with scores of 5, 10, and 15 representing mild, moderate, and severe anxiety symptoms, respectively [57].

#### Perceived Stress Scale

The PSS is designed to measure the degree to which situations in one’s life are appraised as stressful. Among two versions of the PSS—the 14-item version (PSS-14) and the 10-item version (PSS-10)—the PSS-10 was recommended because the four additional items of the PSS-14 show relatively low factor loading [58]. Therefore, the PSS-10 was used in our study. This scale assesses perceived stressful experiences or stress responses over the previous month. Total possible scores range from 0 to 40. Higher scores represent high stress levels [59].

#### World Health Organization Health and Work Performance Questionnaire

The WHO-HPQ is a self-report instrument designed to estimate the workplace costs of health problems in terms of self-reported sickness absences and reduced job performance (ie, presenteeism). The WHO-HPQ measures presenteeism with the following two questions: “On a scale from 0 to 10, where 0 is the worst job performance anyone could have at your job and 10 is the performance of a top worker, how would you rate the usual performance of most workers in a job similar to yours?” and “Using the same 0-10 scale, how would you rate your overall job performance on the days you worked during the past 4 weeks?” A low presenteeism score indicated poorer performance [60].

#### Multidimensional Assessment of Interoceptive Awareness

Interoceptive awareness has been regarded as an essential factor in meditation and stress reduction. The MAIA was developed as a self-report instrument for experimental interoception research and for assessment of mind-body therapies [61]. It is a 32-item self-report measure that assesses interoceptive awareness on the following eight subscales: noticing, not-distracting, not-worrying, attention regulation, emotional awareness, self-regulation, body listening, and trusting. Each item is assessed on a 6-point Likert scale, ranging from 0 (*never*) to 5 (*always*). Higher scores indicate better interoceptive awareness [62].

#### European Quality of Life Five-Dimension Five-Level Scale

The EQ-5D-5L is a standardized instrument used to measure health-related quality of life [63]. Applicable to a wide range of health conditions and treatments, it provides a simple descriptive profile and a single index value for health status.

### Randomization

**Figure 1:**
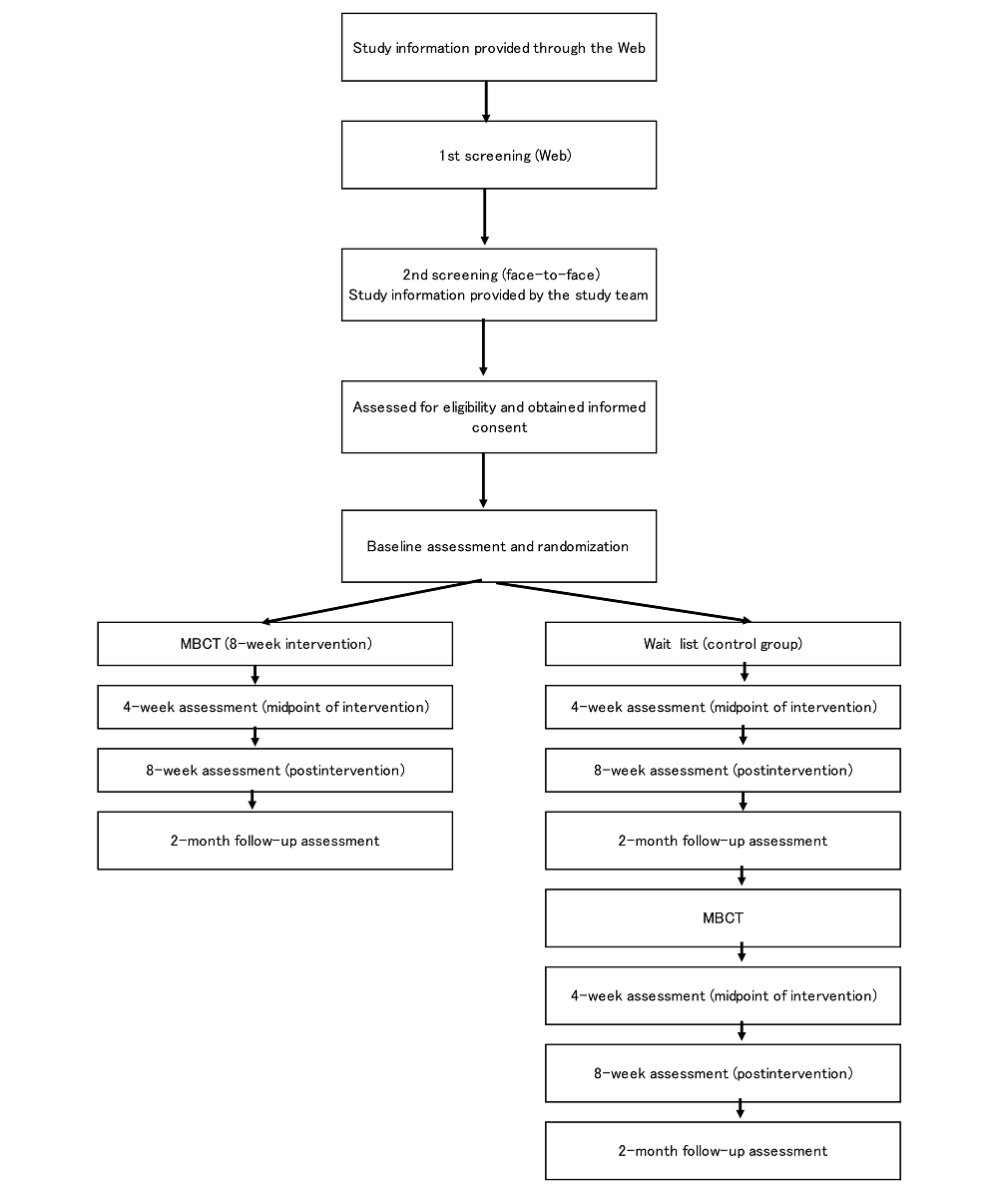
Flowchart of the effectiveness and cost-effectiveness of mindfulness-based cognitive therapy (MBCT) for improving subjective well-being among healthy individuals.

Eligible participants will be randomly assigned, at a 1:1 ratio, to the MBCT group or the wait-list control group. A computer-generated random number stratified by the baseline score of the SWLS will be allocated to each participant. The Keio Center of Clinical Research Project Management Office, which is not associated with this study, will manage the process of the randomization. The flow diagram of the study participants is shown in Figure 1.

### Blinding

Due to the nature of this psychological intervention, the randomization status of participants and program instructors cannot be blinded. Because all measures obtained through the study period are self-reported, there will be no assessors to judge the state of participants.

### Intervention and Control Groups

#### Mindfulness-Based Cognitive Therapy Group

The intervention program used in the study is the modified version of MBCT based on the book *Mindfulness: A Practical Guide to Finding Peace in a Frantic World* [64]. This program has been developed by modifying an MBCT program to improve the well-being of a nonclinical population. The contents of the program are shown in Table 1. The main differences of this program from MBCT are that (1) the lecture relevant to depression will be skipped and (2) compassion meditation and activity records (ie, pleasant, unpleasant, appreciation events, and nourishing and depriving activities) will be introduced. In the program, participants will learn both cognitive approaches and mindfulness practices (eg, raisin exercise, body scan, sitting meditation, mindful walking, and three-step breathing space).

The program will consist of eight weekly sessions. Each session will be in a group format—15 participants at the most—and will last for 2 hours. The participants will be asked to practice mindfulness meditation for 30-60 minutes as their daily homework and to keep a record of the type of meditation and the amount of time they practiced.

The first author (MS) will lead the sessions as the principal instructor. Dr Sado has been qualified to teach MBSR through a program at the University of Massachusetts, Boston, USA; is on the training path for MBCT teachers at Oxford University, Oxford, UK; and has 9 years of experience in mindfulness practice. The second (TK) and third (AN) authors will join the course as assistant instructors.

#### Control Group

Participants on the wait list will have no interventions during the intervention period. They will be asked not to take part in other mindfulness or meditation activities. After the first intervention term is completed, the participants in the control group will be given an opportunity to attend the MBCT program.

**Table 1:** Contents of the intervention program.

Session	Theme	Contents
1	Waking up to the automatic pilot	Psychoeducation: What is mindfulness? Exercise: Mindful eating (ie, *raisin exercise*), asking yourself why you are here now, and mindfulness of body and breath Homework: Mindfulness of body and breath, mindfulness of a routine activity, and let go of habits
2	Keeping the body in mind	Psychoeducation: Association of mood and thoughts Exercise: Mindfulness of body and breath, thoughts and feelings exercise, and body scan Homework: Body scan, pleasant event calendar, mindfulness in everyday life, and let go of habits
3	The mouse in the maze	Psychoeducation: Awareness of mind wandering and focusing on the breath Exercise: Breathing meditation, meditation of sounds, gentle yoga, and mindful walking Homework: Three-step breathing space, gentle yoga, mindful walking, diary of appreciation and gratitude events, and let go of habits
4	Moving beyond the rumor mill	Psychoeducation: Staying present Exercise: Mindfulness meditations (ie, breathing as well as sounds and thoughts) Homework: Mindfulness meditations (ie, breathing, sounds and thoughts, and three-step breathing space), unpleasant events calendar, and let go of habits
5	Turning toward difficulties	Psychoeducation: Exploring difficulty Exercise: Mindfulness meditations (ie, breathing, sounds and thoughts, and exploring difficulty) Homework: Mindfulness meditations (ie, breathing, sounds and thoughts, exploring difficulty, and three-step breathing space) and let go of habits
6	Trapped in the past or living in the present	Psychoeducation: Cognitive biases and compassion for myself Exercise: Mindfulness meditations, compassion meditation, and watching the movie *Happy* about subjective well-being Homework: Mindfulness meditations (ie, sounds and thoughts, exploring difficulty, compassion, and three-step breathing space) and diary of your kind behavior
7	When did you stop dancing?	Psychoeducation: Choosing functional behaviors, behavioral activation, and identifying triggers Exercise: Mindfulness meditations (ie, breathing as well as sounds and thoughts) Homework: Mindfulness meditations (ie, choose what you like and three-step breathing space) and diary of activity that nourishes you
8	Your wild and precious life	Personal reflections of the course, plans for future practice, strategies for maintaining momentum, and farewell Exercise: Body scan and asking yourself why you are here now and what you realized through the program

### Outcomes

#### Primary Outcome

The primary outcome is the difference in mean change scores between the baseline and postintervention assessments on the SWLS for the MBCT group as compared to the control group.

#### Secondary Outcomes

The secondary outcomes are the differences in mean change scores between the baseline and postintervention assessments on the FS, SPANE, RSES, FFMQ, CD-RISC, SCS, QIDS, GAD-7, PSS, WHO-HPQ, MAIA, and EQ-5D-5L for the MBCT group as compared to the control group.

### Cost-Effectiveness

Cost-effectiveness will be assessed based on the incremental cost-effectiveness ratio that represents the incremental cost divided by the incremental effectiveness between the groups. With respect to cost, we only include the human resource cost to deliver the sessions, since the study targets healthy individuals. Because the population targeted in the study is composed of healthy individuals, incremental effectiveness will be evaluated primarily using the measures of subjective well-being, such as the SWLS. However, we will also use the quality-adjusted life years mapped from the results of the EQ-5D-5L and so on, representing health-related quality of life as the secondary incremental effectiveness outcome. The analyses will be conducted from a third-party payers’ perspective.

### Schedule of Visits and Assessments

All participants will be asked to complete these psychological self-reporting measures at 4 weeks (ie, the intervention midpoint), 8 weeks (ie, postintervention), and 2 months (ie, 16 weeks) after the completion of the intervention, as well as at their baseline assessments (ie, week 0). The assessment schedule is shown in Table 2.

**Table 2:** Schedule of assessments.

Assessment	First screening	Week^a^										
		0	1	2	3	4	5	6	7	8	12	16
Screening (Web)	X											
Screening (face-to-face interview)		X										
Informed consent		X										
Randomization		X										
Mindfulness-based cognitive therapy (MBCT) class			X	X	X	X	X	X	X	X	X	X
Demographics	X	X										
Structured Clinical Interview for the Diagnostic and Statistical Manual of Mental Disorders, Fourth Edition (SCID)		X										
Satisfaction With Life Scale (SWLS)		X				X				X		X
Flourishing Scale (FS)		X				X				X		X
Scale of Positive and Negative Experience (SPANE)		X				X				X		X
Rosenberg Self-Esteem Scale (RSES)		X				X				X		X
Five Facet Mindfulness Questionnaire (FFMQ)		X				X				X		X
Connor-Davidson Resilience Scale (CD-RISC)		X				X				X		X
Self-Compassion Scale (SCS)		X				X				X		X
16-item Quick Inventory of Depressive Symptomatology (QIDS)		X				X				X		X
Generalized Anxiety Disorder 7-item scale (GAD-7)		X				X				X		X
Perceived Stress Scale (PSS)		X				X				X		X
World Health Organization Heath and Work Performance Questionnaire (WHO-HPQ)		X				X				X		X
Multidimensional Assessment of Interoceptive Awareness (MAIA)		X				X				X		X
European Quality of Life Five-Dimension Five-Level Scale (EQ-5D-5L)		X				X				X		X

^a^Psychological self-reporting measures will be completed at baseline (week 0), the intervention midpoint (week 4), postintervention (week 8), and 2 months after the completion of the intervention (week 16).

### Sample Size

We performed sample size calculation based on the results of a previous feasibility study that we had conducted, which assessed the feasibility, safety, and effectiveness of MBCT for improving subjective well-being with a single arm. The pre-post difference in the mean score of the SWLS in the study was 3.1 (SD 3.4). With a statistical power of at least 80% and a two-sided 5% significance level, the sample size was calculated to be 20 participants for each arm. Allowing for a dropout rate of approximately 20%, we determined that each arm would need 25 participants, for a total of 50 participants.

### Statistical Analysis

A 5% significance level will be used for all statistical analyses. To compare differences in the baseline demographics and clinical characteristics of the two groups, unpaired *t* tests will be used for the continuous variables and chi-square tests for the categorical variables. The primary and secondary outcomes will be analyzed using an intent-to-treat approach and a mixed-effect model repeat measurement, a method of handling dropouts in longitudinal clinical trials. Stata 14 (StataCorp) will be used to carry out statistical analysis.

### Adverse Events

When we notice serious adverse events, we will report them to the Ethics Review Committee of the Keio University School of Medicine.

### Ethics

The authors assert that all procedures contributing to this work comply with the ethical standards of the relevant national and institutional committees on human experimentation and with the Helsinki Declaration of 1975, as revised in 2008. All procedures involving human participants and patients were approved by the Ethics Review Committee of the Keio University School of Medicine (reference number: 20170258). The study has been registered in the University Hospital Medical Information Network (UMIN) Clinical Trials Registry (UMIN 000031885).

### Dissemination

The results of the study will be disseminated at several academic conferences and as published articles in peer-reviewed journals. The study will be implemented and reported in line with the CONSORT (Consolidated Standards Of Reporting Trials) statement.

### Availability of Data and Materials

The datasets are available from the corresponding author upon reasonable request.

## Results

This study began recruiting participants in July 2018 and recruitment was completed at the end of September 2019. Data collection and dataset construction was completed by the end of March 2020.

## Discussion

This study aims to investigate the effectiveness of MBCT in the improvement of subjective well-being for healthy individuals in the community. It will attempt to detect meaningful differences in the target outcomes. When we use psychological scales that were developed in a different culture, their validity can become a critical issue because the constructs that the study measures investigate tend to be strongly affected by culture. Therefore, we decided to adopt only scales validated in a Japanese setting. The limitation of this study is that we set the wait-list group as a control group. Of course, we were aware that allocating an attention placebo (eg, relaxation or other form of psychotherapy) would have been an option for detecting the specific effect attributable to MBCT. However, we judged our choice to be acceptable because our aim is to evaluate clinical effectiveness of augmenting typical daily life with MBCT rather than to assess the efficacy of MBCT.

This study is novel in terms of its assessment of all three aspects of subjective well-being (ie, cognitive, affective, and eudemonic aspects) in the absence of other such existing works. Subjective well-being has attracted attention because there are indications that better subjective well-being works as a protective factor for better health status, including mental health. Therefore, we believe this study will generate fruitful knowledge for future research in the field.

## Abbreviations

CD-RISC: Connor-Davidson Resilience Scale
DSM-IV: Diagnostic and Statistical Manual of Mental Disorders, Fourth edition
EQ-5D-5L: European Quality of Life Five-Dimension Five-Level Scale
FFMQ: Five Facet Mindfulness Questionnaire
FS: Flourishing Scale
GAD-7: Generalized Anxiety Disorder 7-item scale
GDP: gross domestic product
Keio CSR: Center for Stress Research at Keio University
MAIA: Multidimensional Assessment of Interoceptive Awareness
MBCT: mindfulness-based cognitive therapy
MBI: mindfulness-based intervention
MBSR: mindfulness-based stress reduction
OECD: Organisation of Economic Co-operation and Development
PSS: Perceived Stress Scale
PSS-10: 10-item version of the Perceived Stress Scale
PSS-14: 14-item version of the Perceived Stress Scale
QIDS: 16-item Quick Inventory of Depressive Symptomatology
RSES: Rosenberg Self-Esteem Scale
SCS: Self-Compassion Scale
SPANE: Scale of Positive and Negative Experience
SWLS: Satisfaction With Life Scale
UMIN: University Hospital Medical Information Network
WHO-HPQ: World Health Organization Health and Work Performance Questionnaire

## References

[1] Stiglitz J, Sen A, Fitoussi J (2010). MIS-Measuring Our Lives.

[2] OECD (2013). OECD Guidelines on Measuring Subjective Well-Being.

[3] (2019). Measuring National Well-Being in the UK: International Comparisons, 2019.

[4] (2009). GDP and Beyond: Measuring Progress in a Changing World.

[5] (2016). A Compass Towards a Just and Harmonious Society: 2015 GNH Survey Report.

[6] The Commission on Measuring Well-Being (2011). Measuring National Well-Being: Proposed Well-Being Indicators.

[7] The Federal Government (2017). Government Report on Wellbeing in Germany.

[8] Huppert FA, Marks N, Clark A, Siegrist J, Stutzer A, Vittersø J, Wahrendorf M (2008). Measuring well-being across Europe: Description of the ESS Well-being Module and preliminary findings. Soc Indic Res.

[9] Steptoe A (2007). Depression and Physical Illness.

[10] Lyubomirsky S, King L, Diener E (2005). The benefits of frequent positive affect: Does happiness lead to success?. Psychol Bull.

[11] Howell RT, Kern ML, Lyubomirsky S (2007). Health benefits: Meta-analytically determining the impact of well-being on objective health outcomes. Health Psychol Rev.

[12] Diener E, Chan M (2011). Happy people live longer: Subjective well-being contributes to health and longevity. Appl Psychol Health Well Being.

[13] Burton CM, King LA (2004). The health benefits of writing about intensely positive experiences. J Res Pers.

[14] Green LS, Oades LG, Grant AM (2006). Cognitive-behavioral, solution-focused life coaching: Enhancing goal striving, well-being, and hope. J Posit Psychol.

[15] Peters ML, Flink IK, Boersma K, Linton SJ (2010). Manipulating optimism: Can imagining a best possible self be used to increase positive future expectancies?. J Posit Psychol.

[16] Khoury B, Lecomte T, Fortin G, Masse M, Therien P, Bouchard V, Chapleau M, Paquin K, Hofmann SG (2013). Mindfulness-based therapy: A comprehensive meta-analysis. Clin Psychol Rev.

[17] Teasdale JD, Segal ZV, Williams JM, Ridgeway VA, Soulsby JM, Lau MA (2000). Prevention of relapse/recurrence in major depression by mindfulness-based cognitive therapy. J Consult Clin Psychol.

[18] Hoge EA, Bui E, Marques L, Metcalf CA, Morris LK, Robinaugh DJ, Worthington JJ, Pollack MH, Simon NM (2013). Randomized controlled trial of mindfulness meditation for generalized anxiety disorder: Effects on anxiety and stress reactivity. J Clin Psychiatry.

[19] Cohen-Katz J, Wiley SD, Capuano T, Baker DM, Kimmel S, Shapiro S (2005). The effects of mindfulness-based stress reduction on nurse stress and burnout, Part II: A quantitative and qualitative study. Holist Nurs Pract.

[20] de Vibe M, Solhaug I, Tyssen R, Friborg O, Rosenvinge JH, Sørlie T, Bjørndal A (2013). Mindfulness training for stress management: A randomised controlled study of medical and psychology students. BMC Med Educ.

[21] Flook L, Goldberg SB, Pinger L, Bonus K, Davidson RJ (2013). Mindfulness for teachers: A pilot study to assess effects on stress, burnout and teaching efficacy. Mind Brain Educ.

[22] Jain S, Shapiro SL, Swanick S, Roesch SC, Mills PJ, Bell I, Schwartz GE (2007). A randomized controlled trial of mindfulness meditation versus relaxation training: Effects on distress, positive states of mind, rumination, and distraction. Ann Behav Med.

[23] Klatt MD, Buckworth J, Malarkey WB (2009). Effects of low-dose mindfulness-based stress reduction (MBSR-ld) on working adults. Health Educ Behav.

[24] Nyklíček I, Mommersteeg PM, Van Beugen S, Ramakers C, Van Boxtel GJ (2013). Mindfulness-based stress reduction and physiological activity during acute stress: A randomized controlled trial. Health Psychol.

[25] Robins CJ, Keng S, Ekblad AG, Brantley JG (2012). Effects of mindfulness-based stress reduction on emotional experience and expression: A randomized controlled trial. J Clin Psychol.

[26] Shapiro SL, Brown KW, Thoresen C, Plante TG (2011). The moderation of mindfulness-based stress reduction effects by trait mindfulness: Results from a randomized controlled trial. J Clin Psychol.

[27] Shapiro SL, Astin JA, Bishop SR, Cordova M (2005). Mindfulness-based stress reduction for health care professionals: Results from a randomized trial. Int J Stress Manag.

[28] Shapiro SL, Schwartz GE, Bonner G (1998). Effects of mindfulness-based stress reduction on medical and premedical students. J Behav Med.

[29] Song Y, Lindquist R (2015). Effects of mindfulness-based stress reduction on depression, anxiety, stress and mindfulness in Korean nursing students. Nurse Educ Today.

[30] Vieten C, Astin J (2008). Effects of a mindfulness-based intervention during pregnancy on prenatal stress and mood: Results of a pilot study. Arch Womens Ment Health.

[31] Malarkey WB, Jarjoura D, Klatt M (2013). Workplace based mindfulness practice and inflammation: A randomized trial. Brain Behav Immun.

[32] Bartlett L, Lovell P, Otahal P, Sanderson K (2016). Acceptability, feasibility, and efficacy of a workplace mindfulness program for public sector employees: A pilot randomized controlled trial with informant reports. Mindfulness.

[33] Huang S, Li R, Huang F, Tang F (2015). The potential for mindfulness-based intervention in workplace mental health promotion: Results of a randomized controlled trial. PLoS One.

[34] van Dongen JM, van Berkel J, Boot CR, Bosmans JE, Proper KI, Bongers PM, van der Beek AJ, van Tulder MW, van Wier MF (2016). Long-term cost-effectiveness and return-on-investment of a mindfulness-based worksite intervention: Results of a randomized controlled trial. J Occup Environ Med.

[35] Steptoe A, Deaton A, Stone AA (2015). Subjective wellbeing, health, and ageing. Lancet.

[36] Segal ZV, Williams JMG, Teasdale JD (2002). Mindfulness-Based Cognitive Therapy for Depression: A New Approach to Preventing Relapse.

[37] American Psychiatric Association (2000). Diagnostical and Statistical Manual of Mental Disorders. 4th edition, text revision (DSM-IV-TR).

[38] Kadono T (1994). Development and validation of the Japanese version of the Satisfaction With Life Scale [Article in Japanese]. Proceedings of the 36th Annual Convention of the Japanese Association of Educational Psychology.

[39] Sumi K (2013). Reliability and validity of Japanese versions of the flourishing scale and the scale of positive and negative experience. Soc Indic Res.

[40] Sugiura Y, Sato A, Ito Y, Murakami H (2011). Development and validation of the Japanese version of the Five Facet Mindfulness Questionnaire. Mindfulness.

[41] Mimura C, Griffiths P (2008). A Japanese version of the Perceived Stress Scale: Cross-cultural translation and equivalence assessment. BMC Psychiatry.

[42] Arimitsu K (2014). Development and validation of the Japanese version of the Self-Compassion Scale [Article in Japanese]. Shinrigaku Kenkyu.

[43] Mimura C, Griffiths P (2007). A Japanese version of the Rosenberg Self-Esteem Scale: Translation and equivalence assessment. J Psychosom Res.

[44] Shoji M, Mehling WE, Hautzinger M, Herbert BM (2018). Investigating multidimensional interoceptive awareness in a Japanese population: Validation of the Japanese MAIA-J. Front Psychol.

[45] Tsuchiya A, Ikeda S, Ikegami N, Nishimura S, Sakai I, Fukuda T, Hamashima C, Hisashige A, Tamura M (2002). Estimating an EQ-5D population value set: The case of Japan. Health Econ.

[46] Fujisawa D, Nakagawa A, Tajima M, Sado M, Kikuchi T, Iba M (2010). Cross-cultural adaptation of the Quick Inventory of Depressive Symptomatology, self-report (QIDS-SR) [Article in Japanese]. Jpn J Stress Sci.

[47] Muramatsu K, Miyaoka H, Ueshima K, Muramatsu Y, Fuse K, Yoshimine H (2010). Validation and utility of a Japanese version of the GAD-7 [Article in Japanese]. Proceedings of the 51st Congress of the Japanese Society of Psychosomatic Medicine.

[48] Ito M, Nakajima S, Shirai A, Kim Y (2009). Reliability and validity of the Japanese version of the Conner Davidson Resilience Scale: Consideration for general adults and university students [Article in Japanese]. Proceedings of the 20th Annual Report Meeting of the National Institute of Mental Health, National Center of Neurology and Psychiatry.

[49] Suzuki T, Miyaki K, Sasaki Y, Song Y, Tsutsumi A, Kawakami N, Shimazu A, Takahashi M, Inoue A, Kurioka S, Shimbo T (2014). Optimal cutoff values of WHO-HPQ presenteeism scores by ROC analysis for preventing mental sickness absence in Japanese prospective cohort. PLoS One.

[50] Diener E, Emmons RA, Larsen RJ, Griffin S (1985). The Satisfaction With Life Scale. J Pers Assess.

[51] Diener E, Wirtz D, Tov W, Kim-Prieto C, Choi D, Oishi S, Biswas-Diener R (2009). New well-being measures: Short scales to assess flourishing and positive and negative feelings. Soc Indic Res.

[52] Rosenberg M (1965). Society and the Adolescent Self-Image.

[53] Baer RA, Smith GT, Hopkins J, Krietemeyer J, Toney L (2006). Using self-report assessment methods to explore facets of mindfulness. Assessment.

[54] Connor KM, Davidson JR (2003). Development of a new resilience scale: The Connor-Davidson Resilience Scale (CD-RISC). Depress Anxiety.

[55] Neff KD (2003). The development and validation of a scale to measure self-compassion. Self Identity.

[56] Rush A, Trivedi MH, Ibrahim HM, Carmody TJ, Arnow B, Klein DN, Markowitz JC, Ninan PT, Kornstein S, Manber R, Thase ME, Kocsis JH, Keller MB (2003). The 16-Item Quick Inventory of Depressive Symptomatology (QIDS), clinician rating (QIDS-C), and self-report (QIDS-SR): A psychometric evaluation in patients with chronic major depression. Biol Psychiatry.

[57] Spitzer RL, Kroenke K, Williams JB, Löwe B (2006). A brief measure for assessing generalized anxiety disorder: The GAD-7. Arch Intern Med.

[58] Cohen S, Williamson G, Spacapan S, Oskamp S (1988). Perceived stress in a probability sample of the United States. The Social Psychology of Health: The Claremont Symposium on Applied Social Psychology.

[59] Cohen S, Kamarck T, Mermelstein R (1983). A global measure of perceived stress. J Health Soc Behav.

[60] Kessler RC, Barber C, Beck A, Berglund P, Cleary PD, McKenas D, Pronk N, Simon G, Stang P, Ustun TB, Wang P (2003). The World Health Organization Health and Work Performance Questionnaire (HPQ). J Occup Environ Med.

[61] Mehling WE, Price C, Daubenmier JJ, Acree M, Bartmess E, Stewart A (2012). The Multidimensional Assessment of Interoceptive Awareness (MAIA). PLoS One.

[62] Bornemann B, Herbert BM, Mehling WE, Singer T (2014). Differential changes in self-reported aspects of interoceptive awareness through 3 months of contemplative training. Front Psychol.

[63] Brooks R (1996). EuroQol: The current state of play. Health Policy.

[64] Williams M, Penman D (2011). Mindfulness: A Practical Guide to Finding Peace in a Frantic World.

